# Supplementary material for: Pharmacologic Targeting of MMP2/9 Decreases Peritoneal Metastasis Formation of Colorectal Cancer in a Human Ex Vivo Peritoneum Culture Model
Source: Cancers (Basel). 2022 Aug 2;14(15):3760. doi: 10.3390/cancers14153760 (PMC9367441; doi:10.3390/cancers14153760)
Supplement: Supplementary file 1 [file cancers-14-03760-s001.zip › cancers-1851946_uncropped WB figures.pdf]

Fig. S2 B

MMP2
~65 kDa

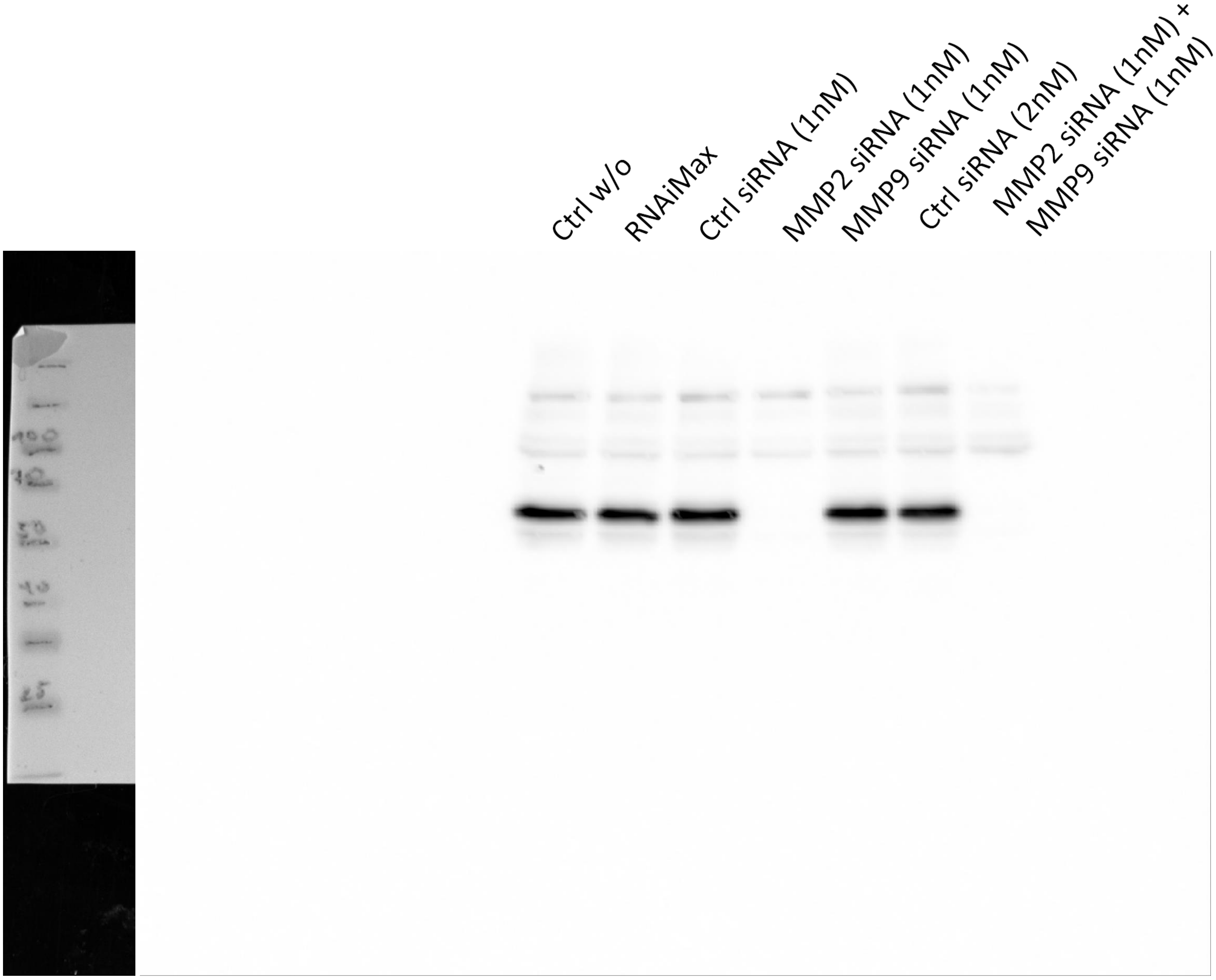

Fig. S2 B

Ctrl w/o
RNAiMax
Ctrl siRNA (1nM)
MMP2 siRNA (1nM)
MMP9 siRNA (1nM)
Ctrl siRNA (2nM)
MMP2 siRNA (1nM) +
MMP9 siRNA (1nM)

MMP9
~110 kDa

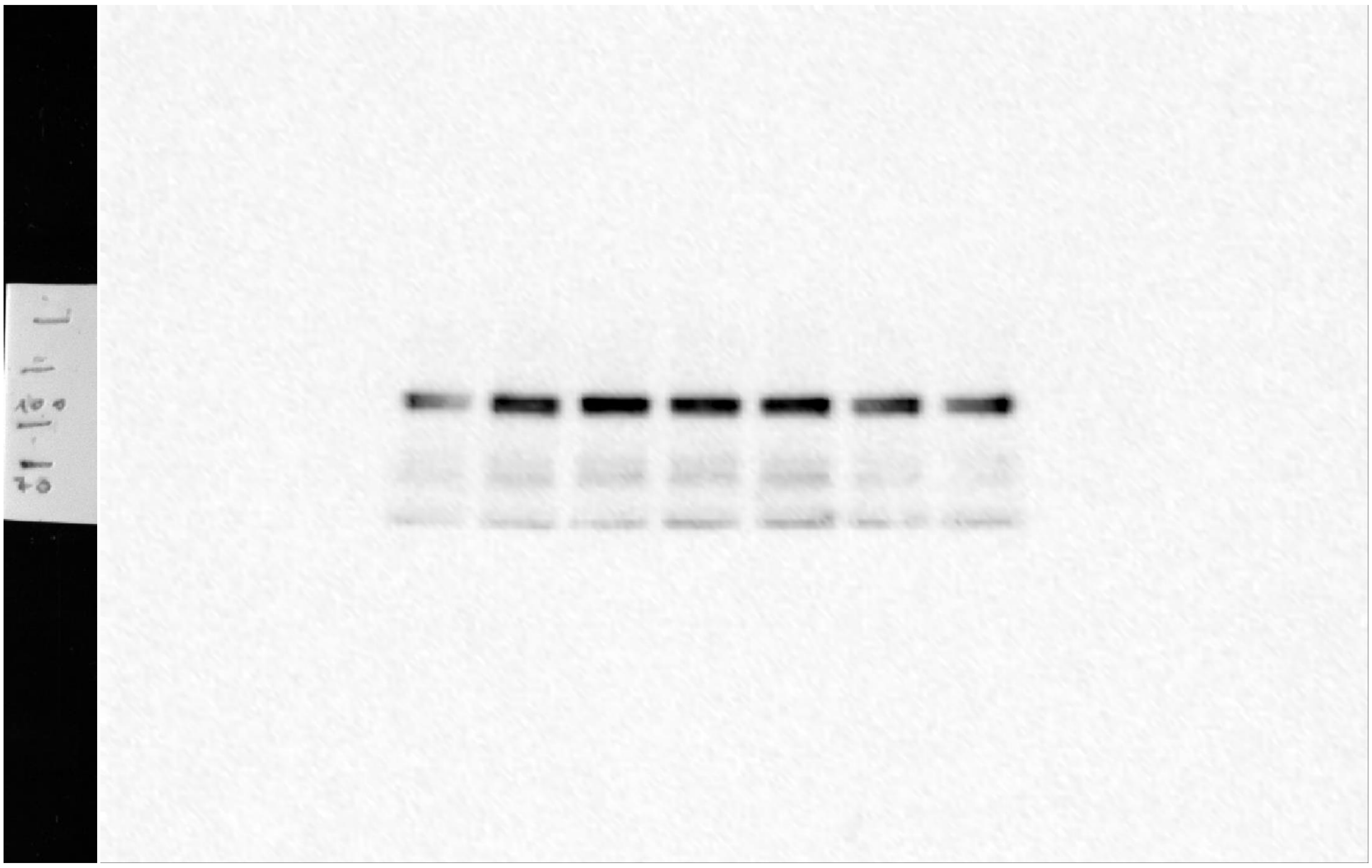

Fig. S2 B

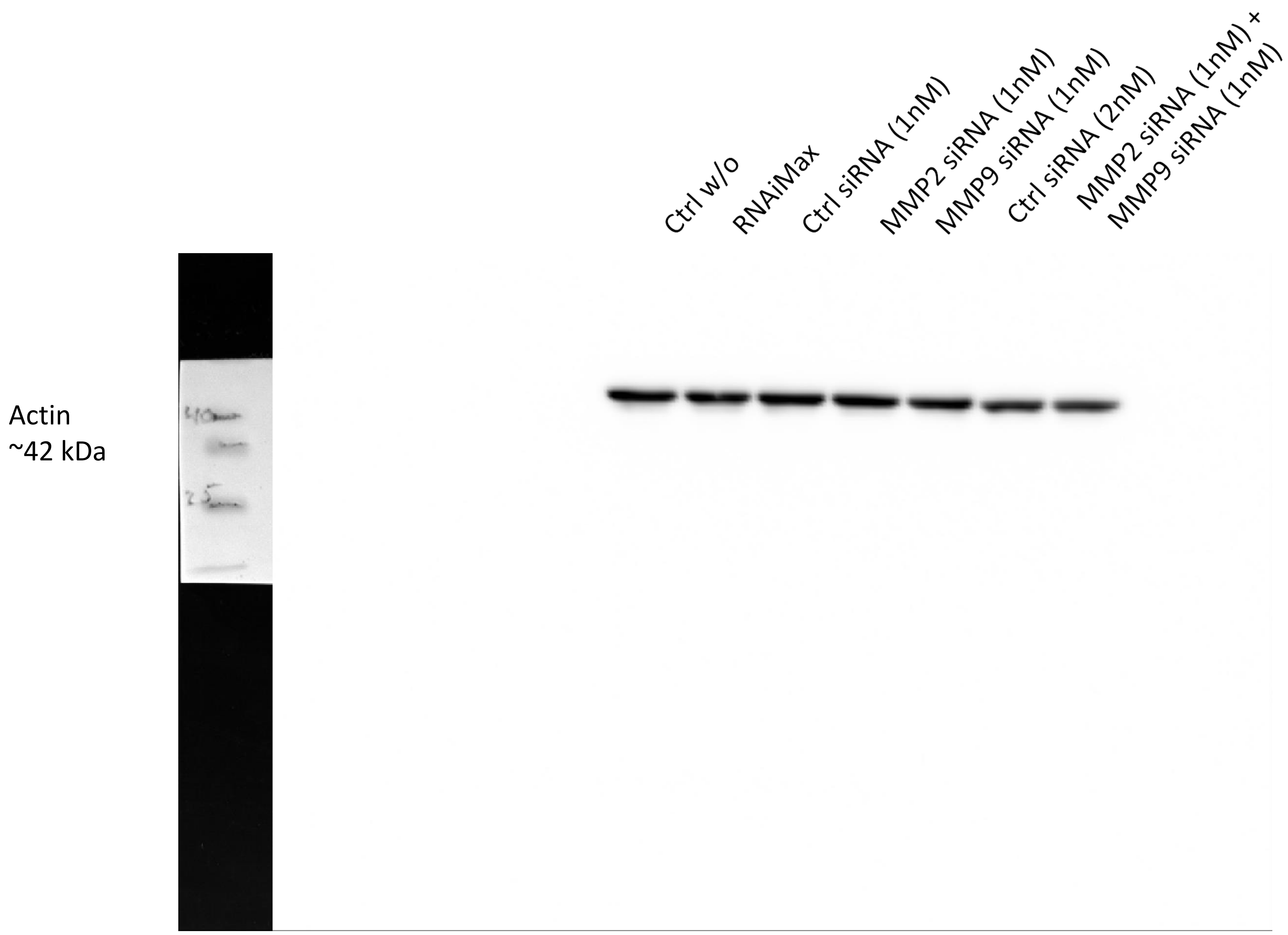
Ctrl w/o
RNAiMax
Ctrl siRNA (1nM)
MMP2 siRNA (1nM)
MMP9 siRNA (1nM)
Ctrl siRNA (2nM)
MMP2 siRNA (1nM) +
MMP9 siRNA (1nM)
Actin
~42 kDa

Fig. S3A

Fibronectin
285kDa

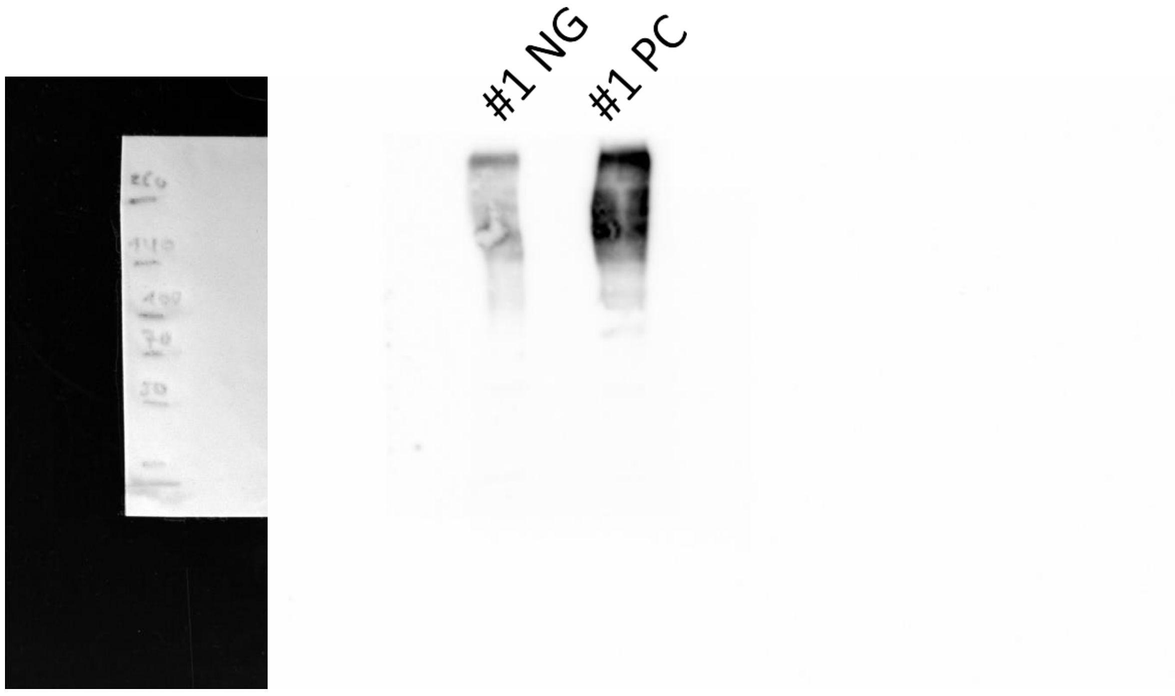

ß-Aktin
42 kDa

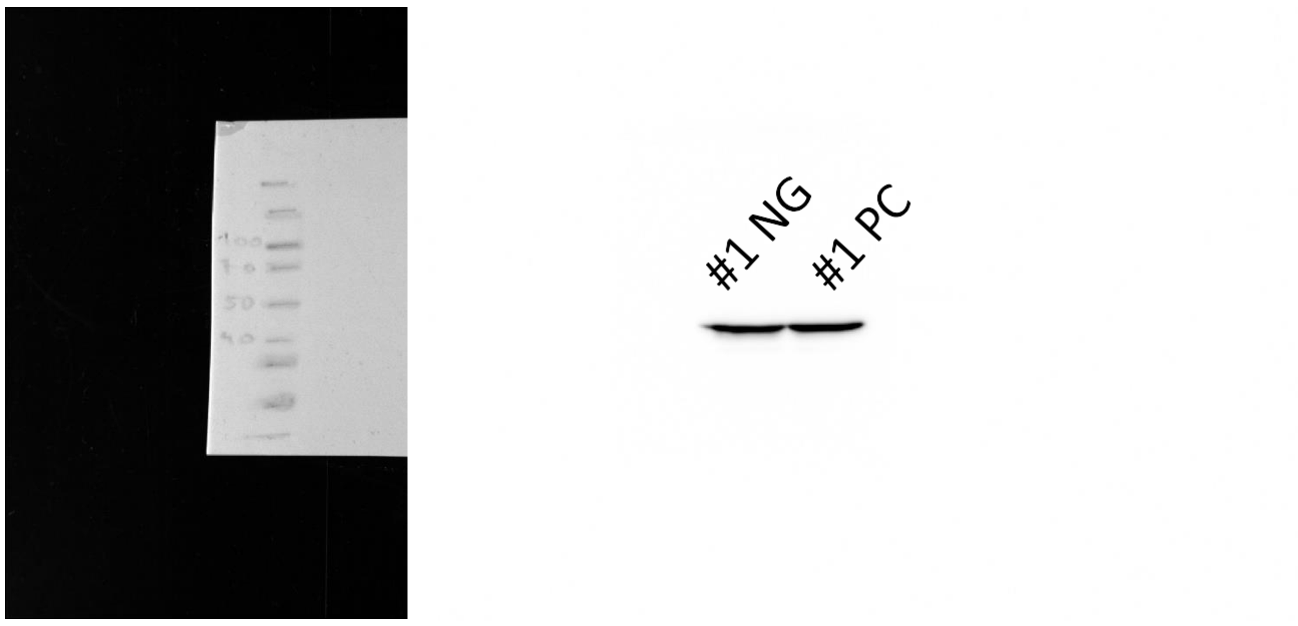

	Ratio (Cleaved Fibronectin/Fibronectin)
#1 NG	1.32
#1 PC	2.08

Fibronectin
285kDa

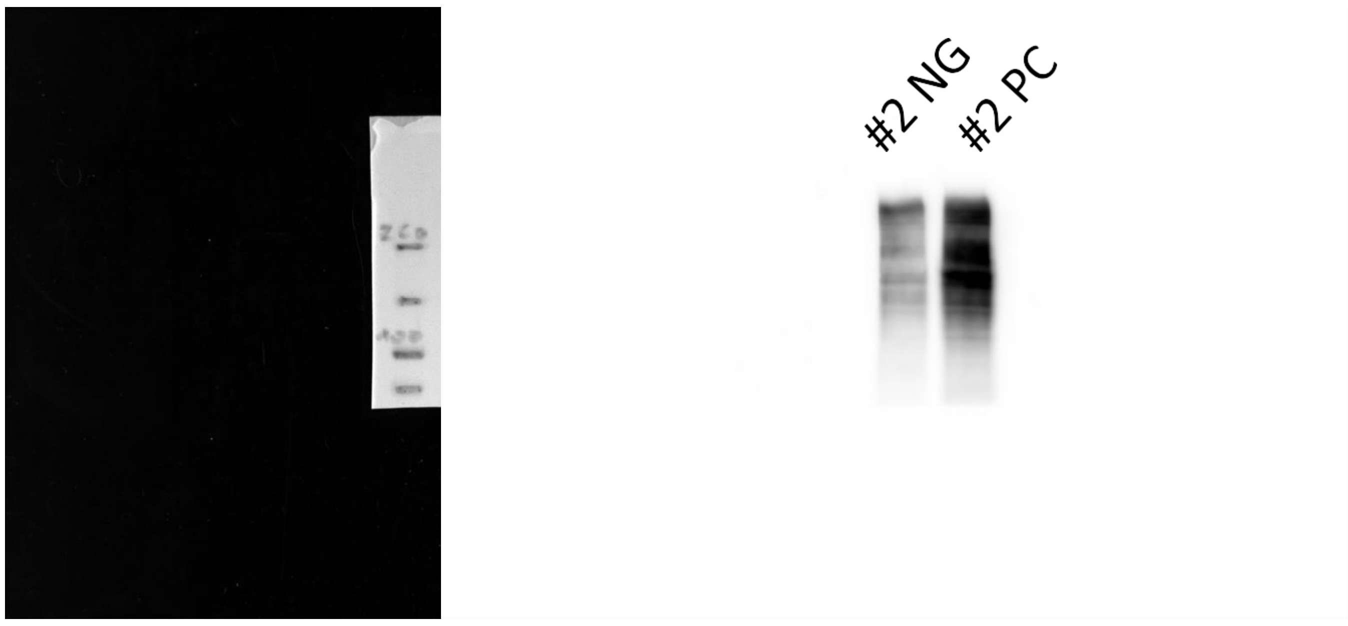

ß-Aktin
42 kDa

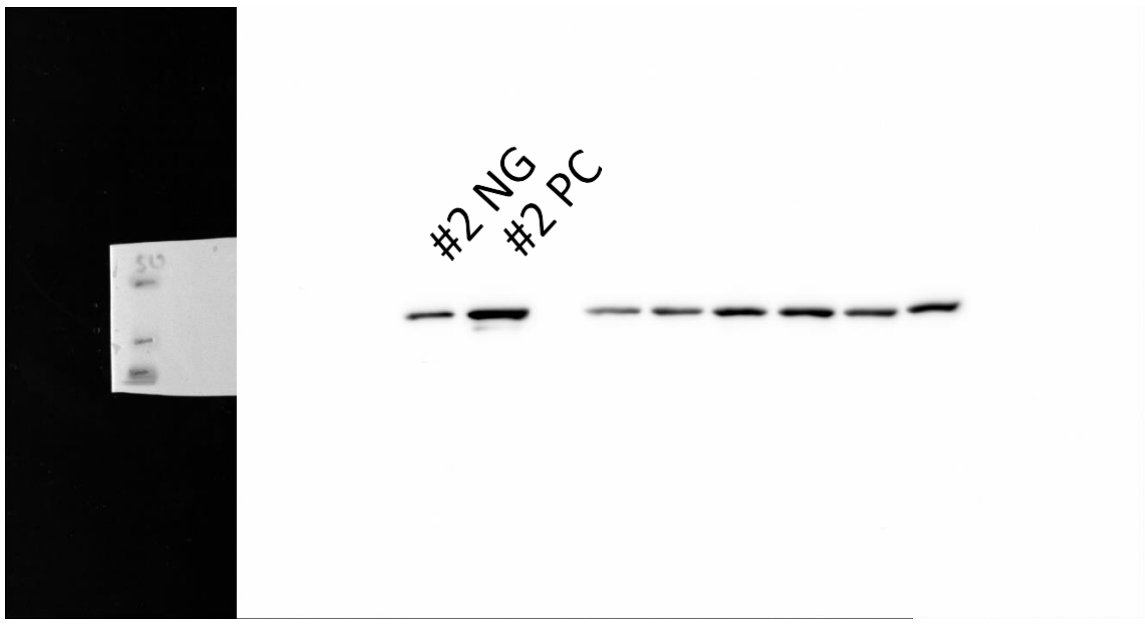

	Ratio (Cleaved Fibronectin/Fibronectin)
#2 NG	1.74
#2 PC	2.11

Fig. S3A

Fibronectin
285kDa

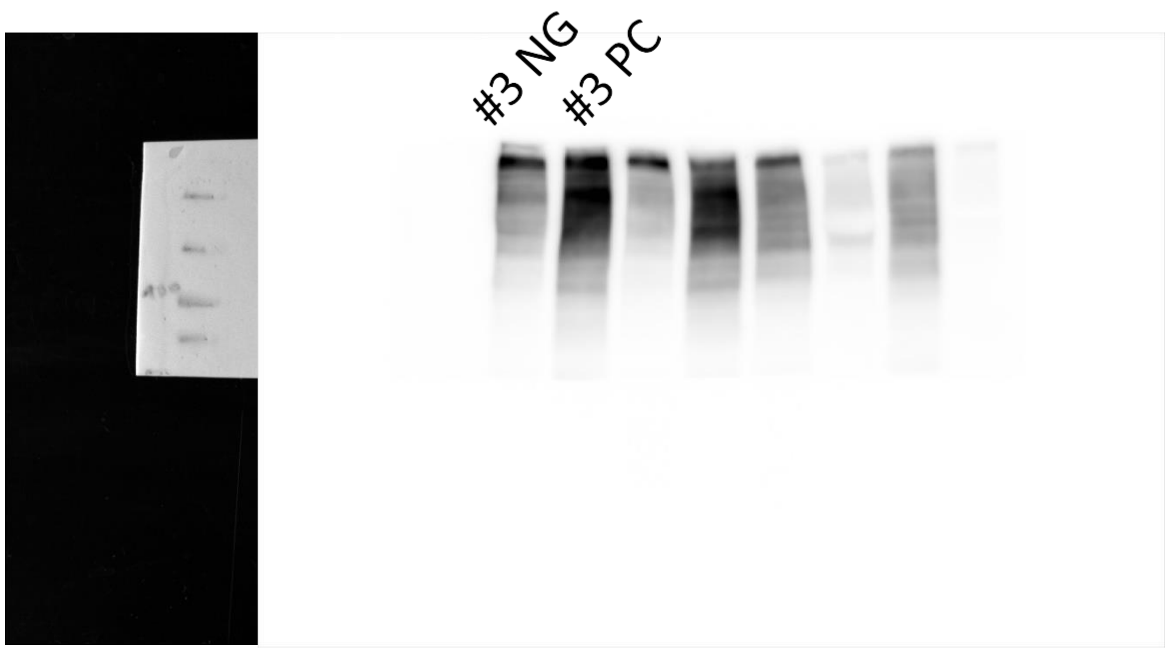

ß-Aktin
42 kDa

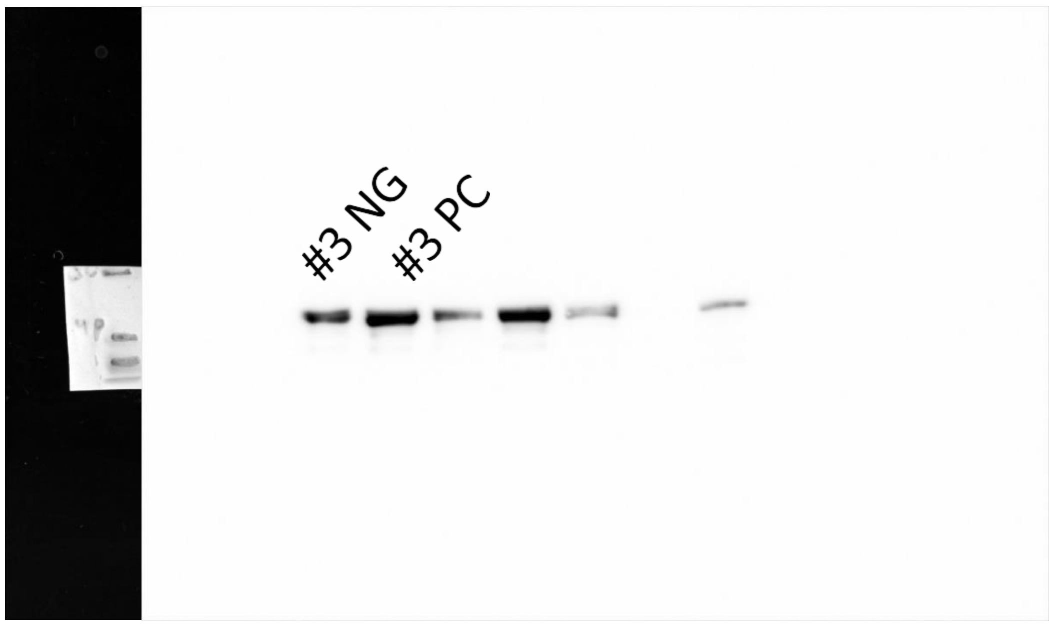

	Ratio (Cleaved Fibronectin/Fibronectin)
#3 NG	0.19
#3 PC	10.23

Fig. S3B

SKOV3

Fibronectin
285 kDa

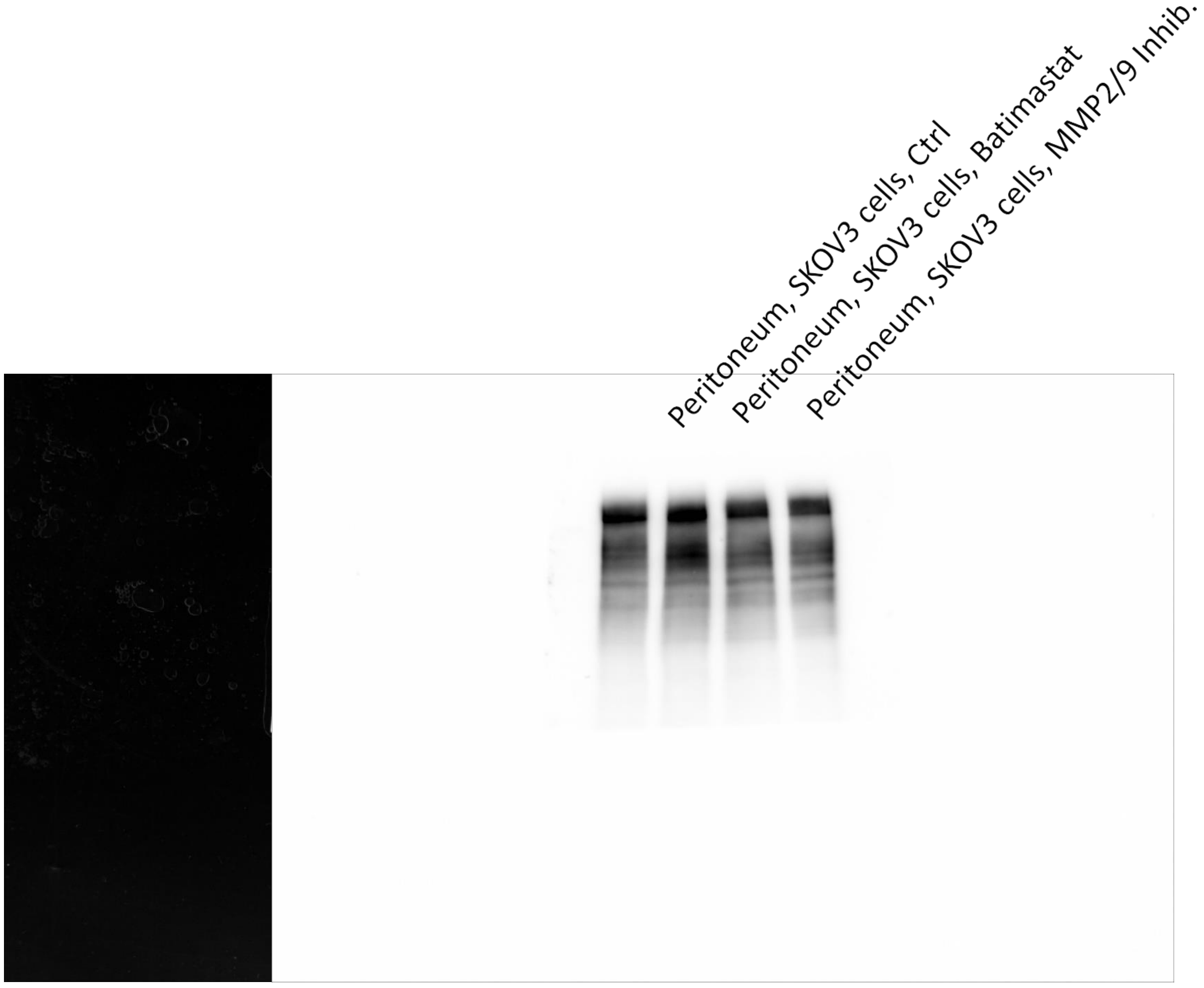

ß-Aktin
42 kDa

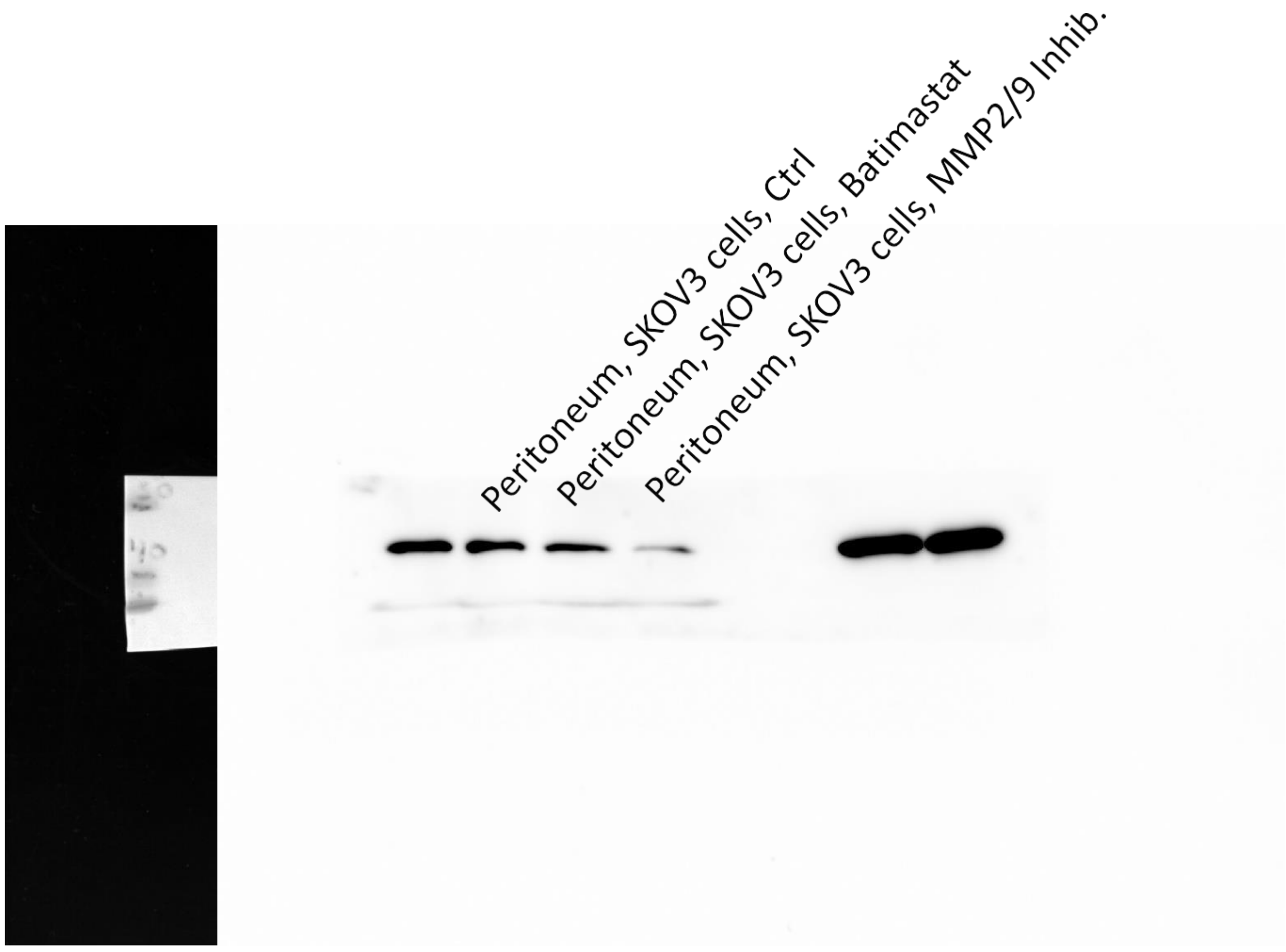

	Ratio (Cleaved Fibronectin/Fibronectin)
P025-21 w SKOV3 Ctrl	1.448697549
P025-21 w SKOV3 + Batimastat	0.860177954
P025-21 w SKOV3 + MMP2/9 inhinbitor	0.905649181

Fig. S3B

SKOV3

Fibronectin
285kDa

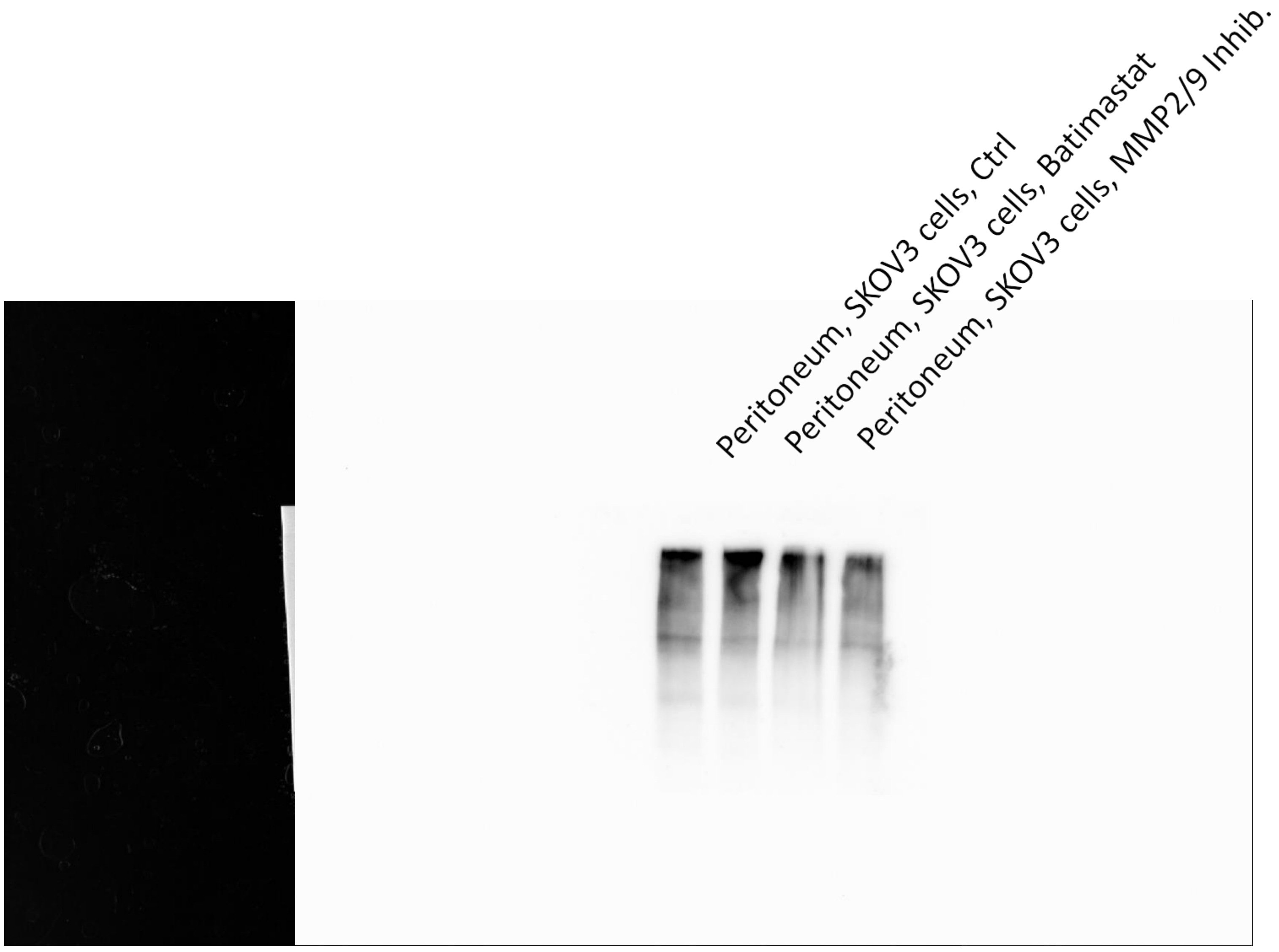

ß-Aktin
42 kDa

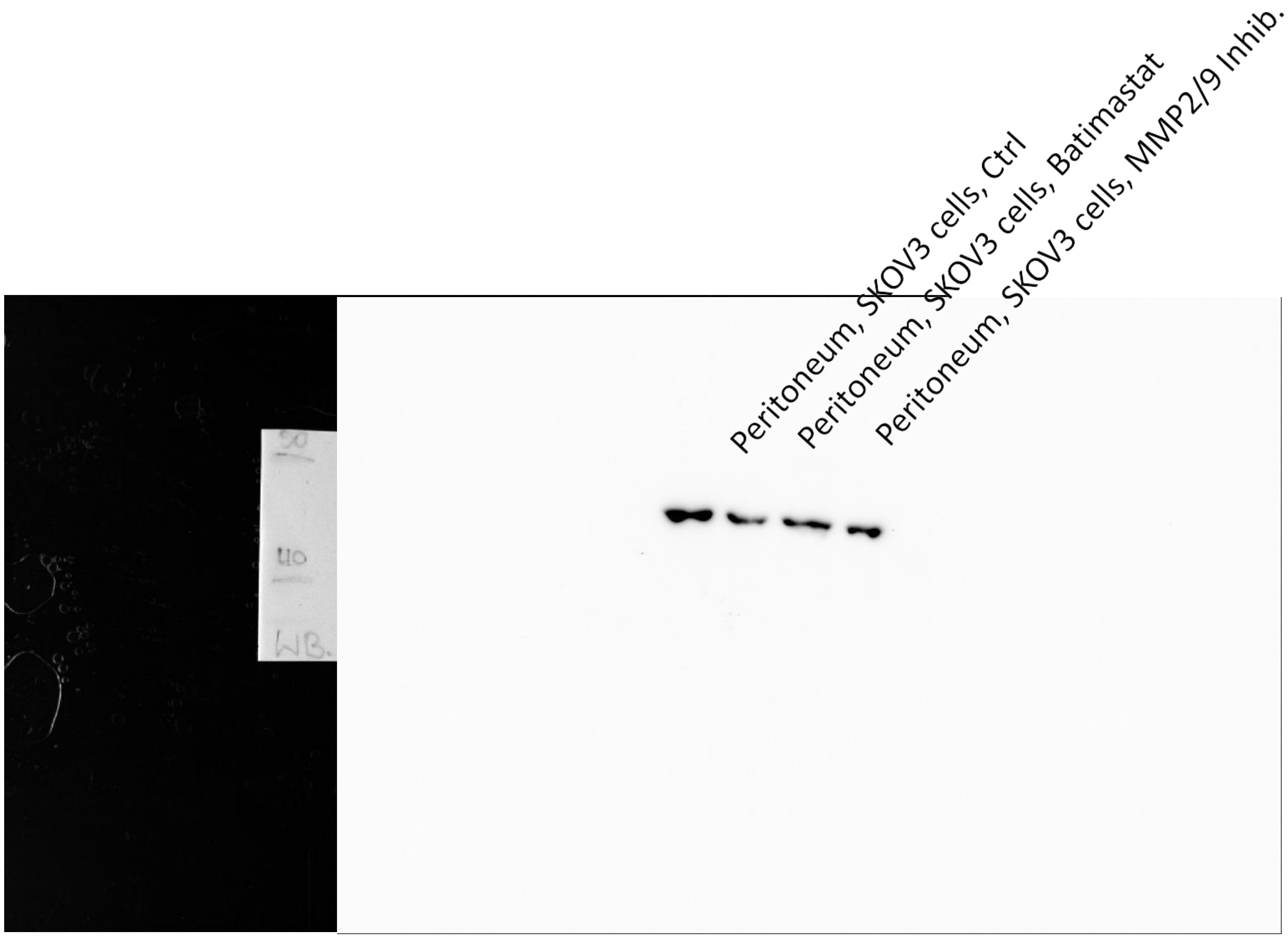

	Ratio (Cleaved Fibronectin/Fibronectin)
P007-23 +SKOV3	1.01
P007-23 +SKOV3 +Batimastat	0.27
P007-23 +SKOV3 +MMP2/9 inhibitor	0.21

Fig. S3B

SKOV3

Fibronectin
285kDa

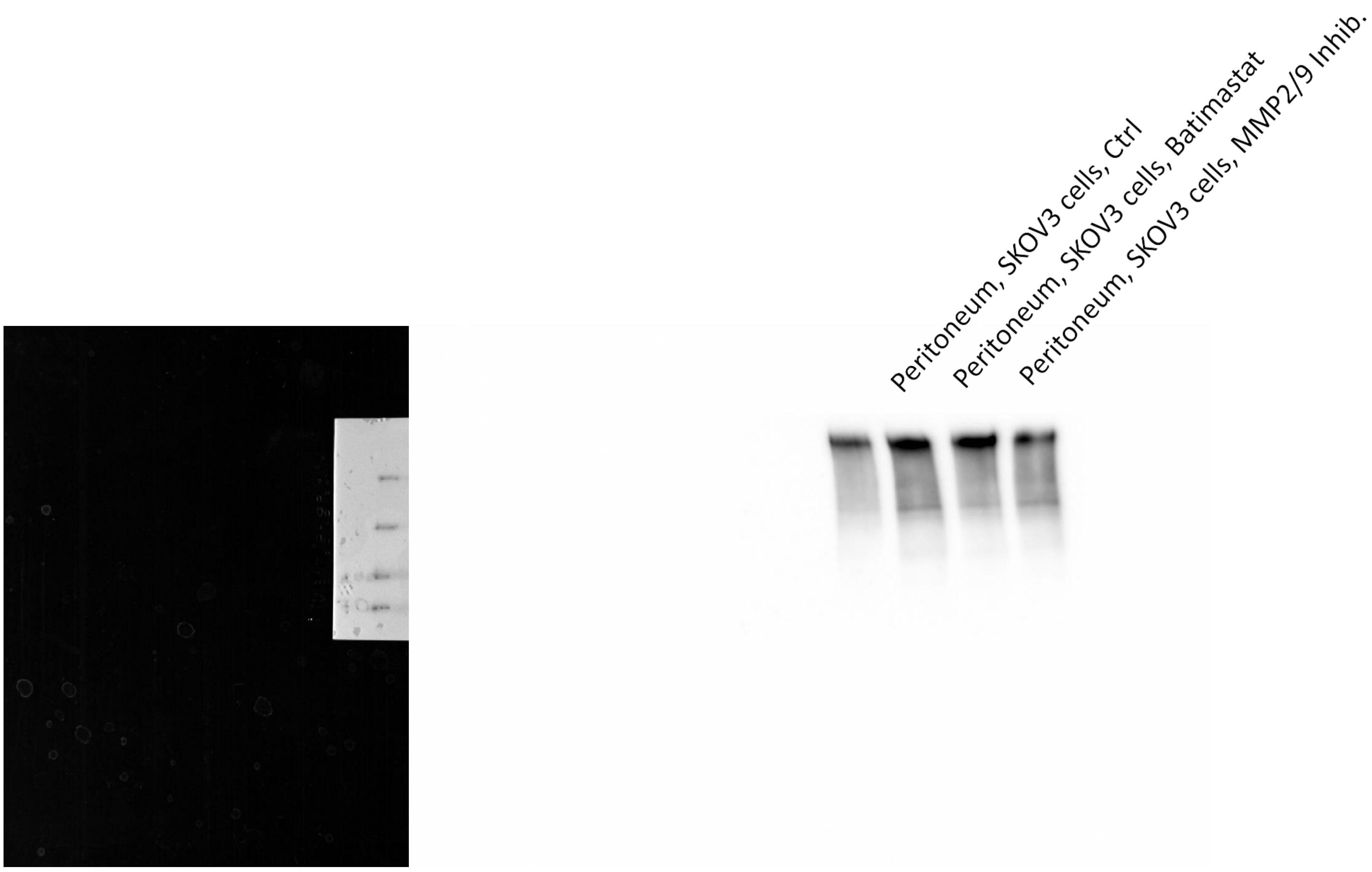

ß-Aktin
42 kDa

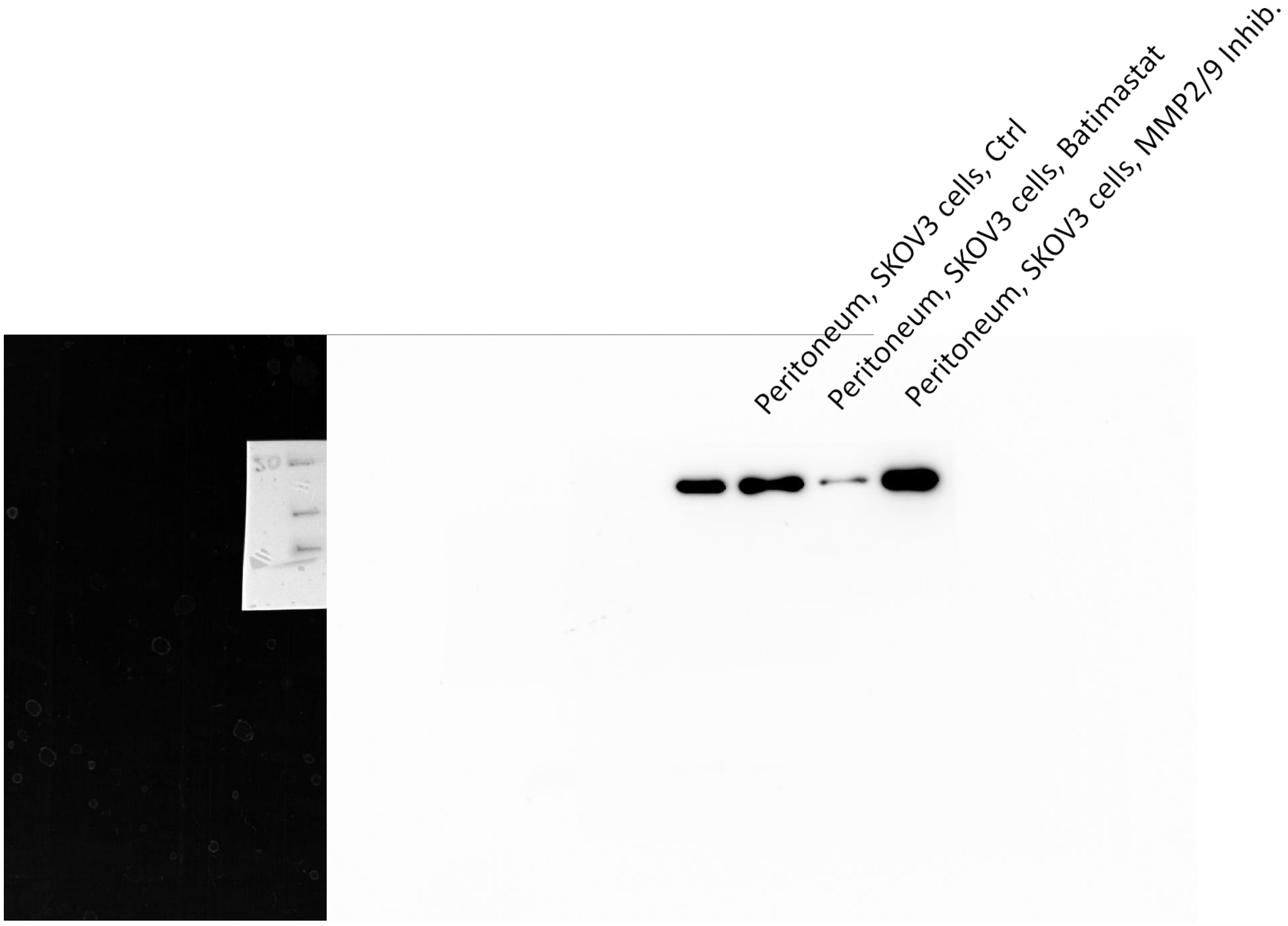

	Ratio (Cleaved Fibronectin/Fibronectin)
P007-23 +SKOV3	0.02
P007-23 +SKOV3 +Batimastat	0.03
P007-23 +SKOV3 +MMP2/9 inhibitor	0.07

Fig. S3B

HT-29

Fibronectin
285kDa

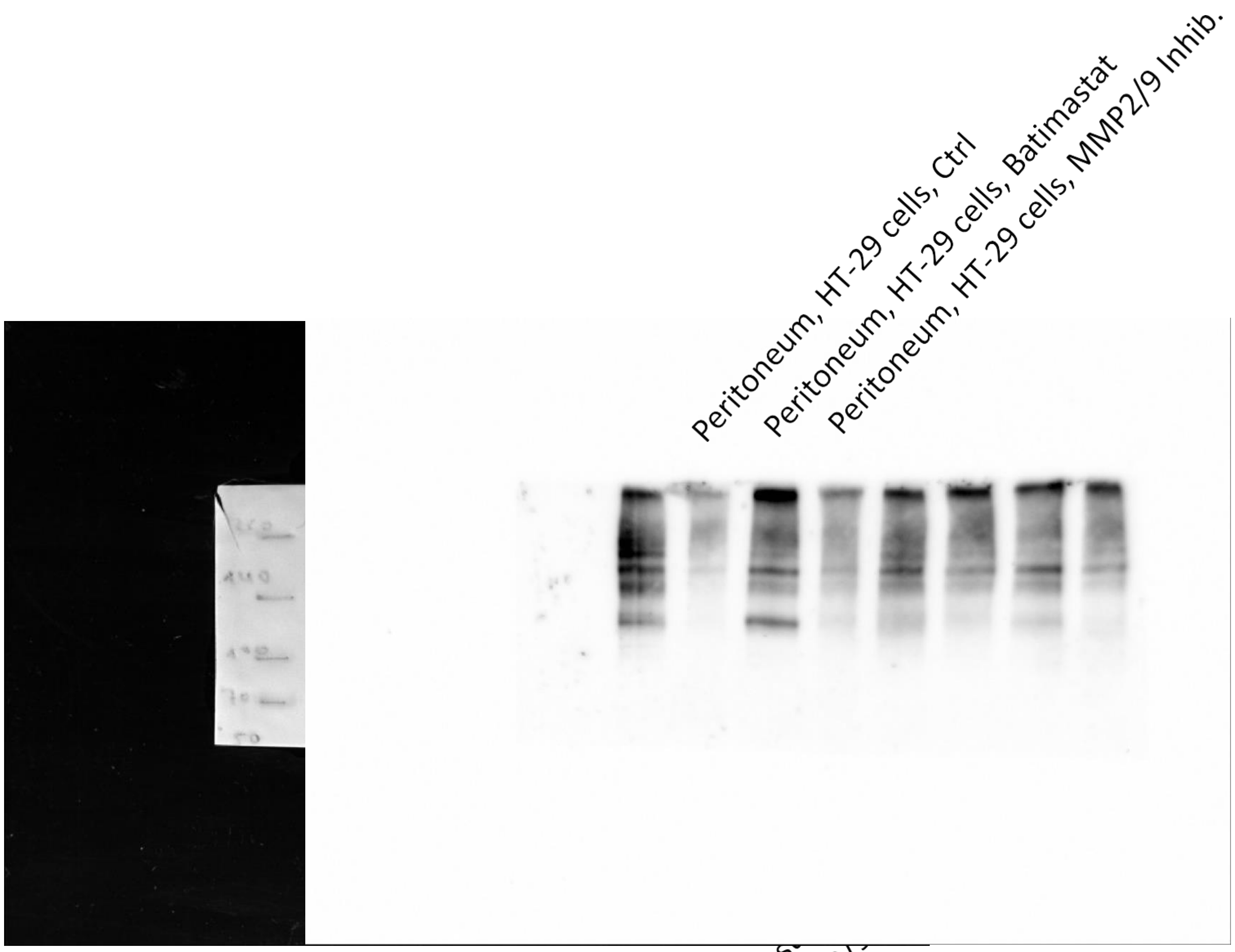

ß-Aktin
42 kDa

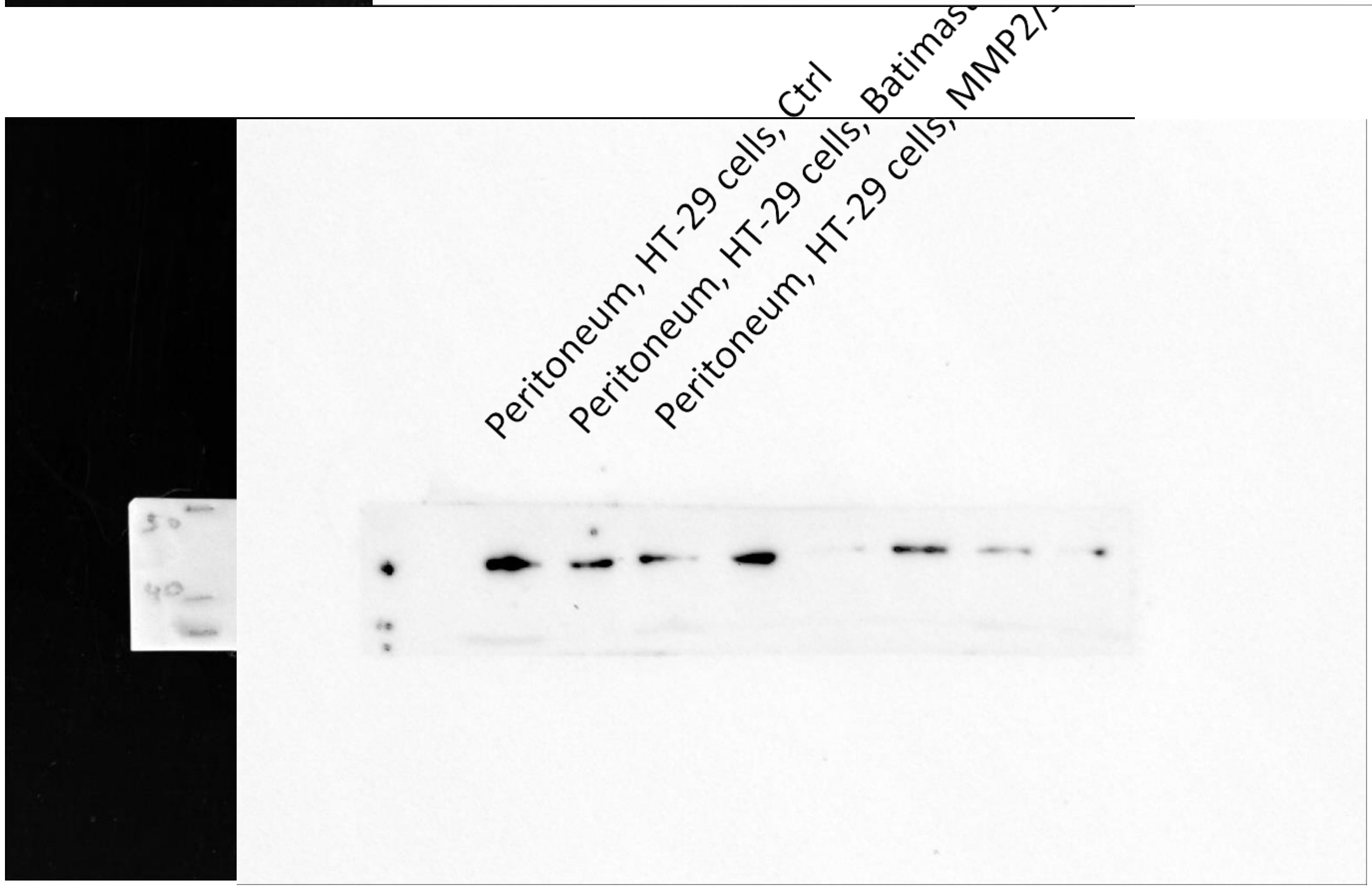

	Ratio (Cleaved Fibronectin/Fibronectin)
P014-22 +HT-29	1.15
P014-22 +HT-29 +Batimastat	1.43
P014-22 +HT-29 +MMP2/9 inhibitor	0.82

Fig. S3B

HT-29

Fibronectin
285kDa

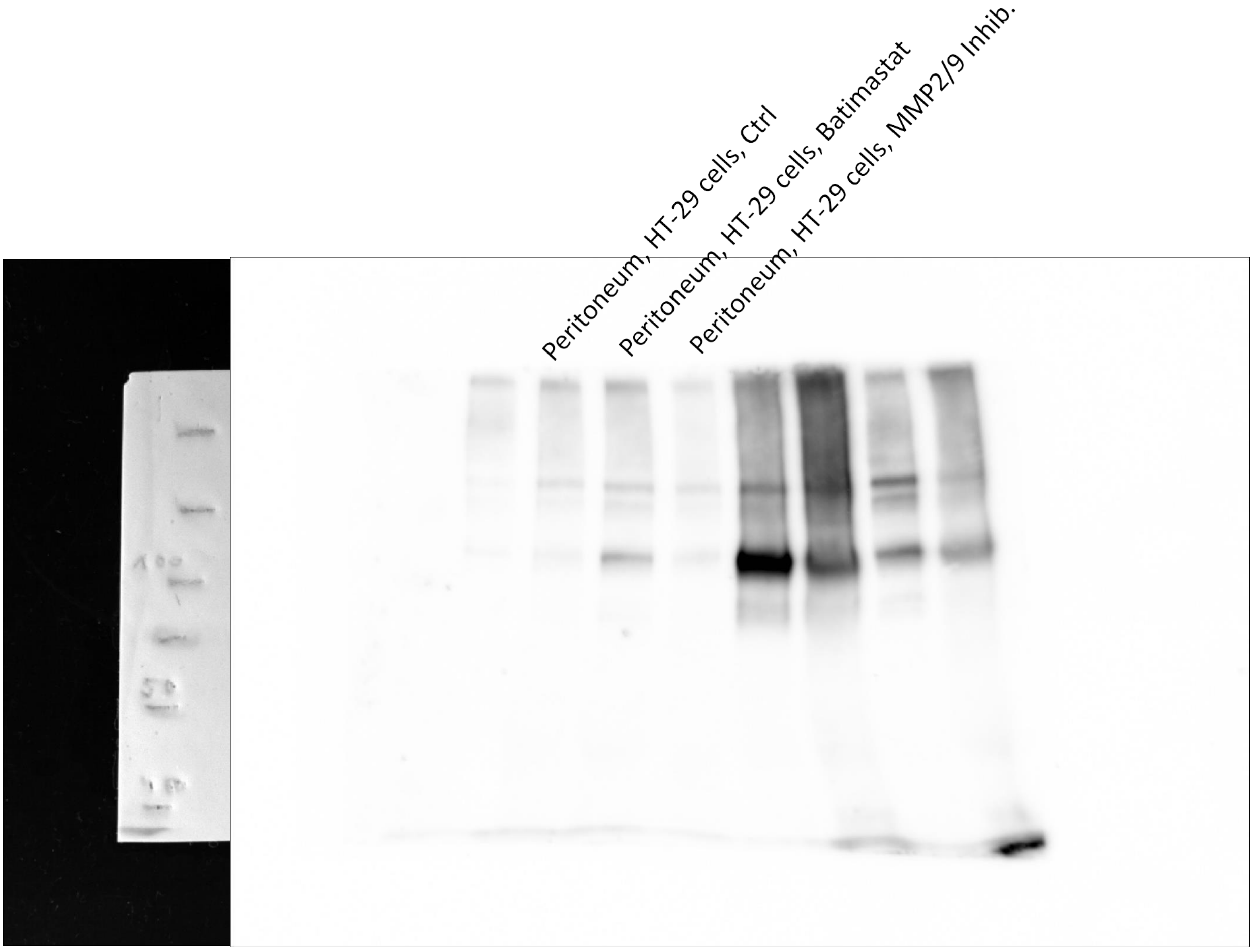

ß-Aktin
42 kDa

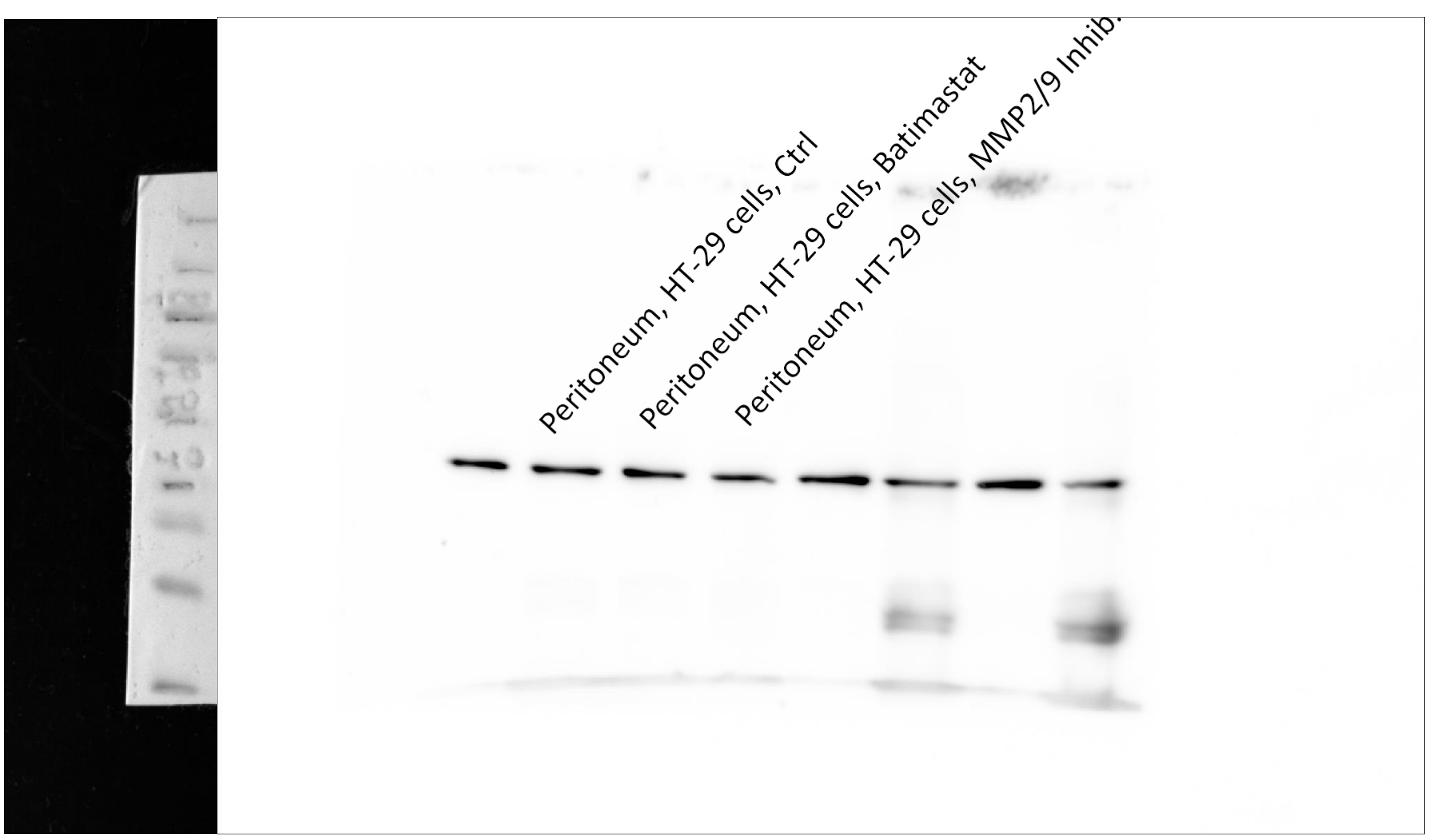

	Ratio (Cleaved Fibronectin/Fibronectin)
P016-22 +HT-29	0.77
P016-22 +HT-29 +Batimastat	2.34
P016-22 +HT-29 +MMP2/9 inhibitor	1.41
