# Supplementary figures and images for: Pharmacologic Targeting of MMP2/9 Decreases Peritoneal Metastasis Formation of Colorectal Cancer in a Human Ex Vivo Peritoneum Culture Model
Source: Cancers (Basel). 2022 Aug 2;14(15):3760. doi: 10.3390/cancers14153760 (PMC9367441; doi:10.3390/cancers14153760)

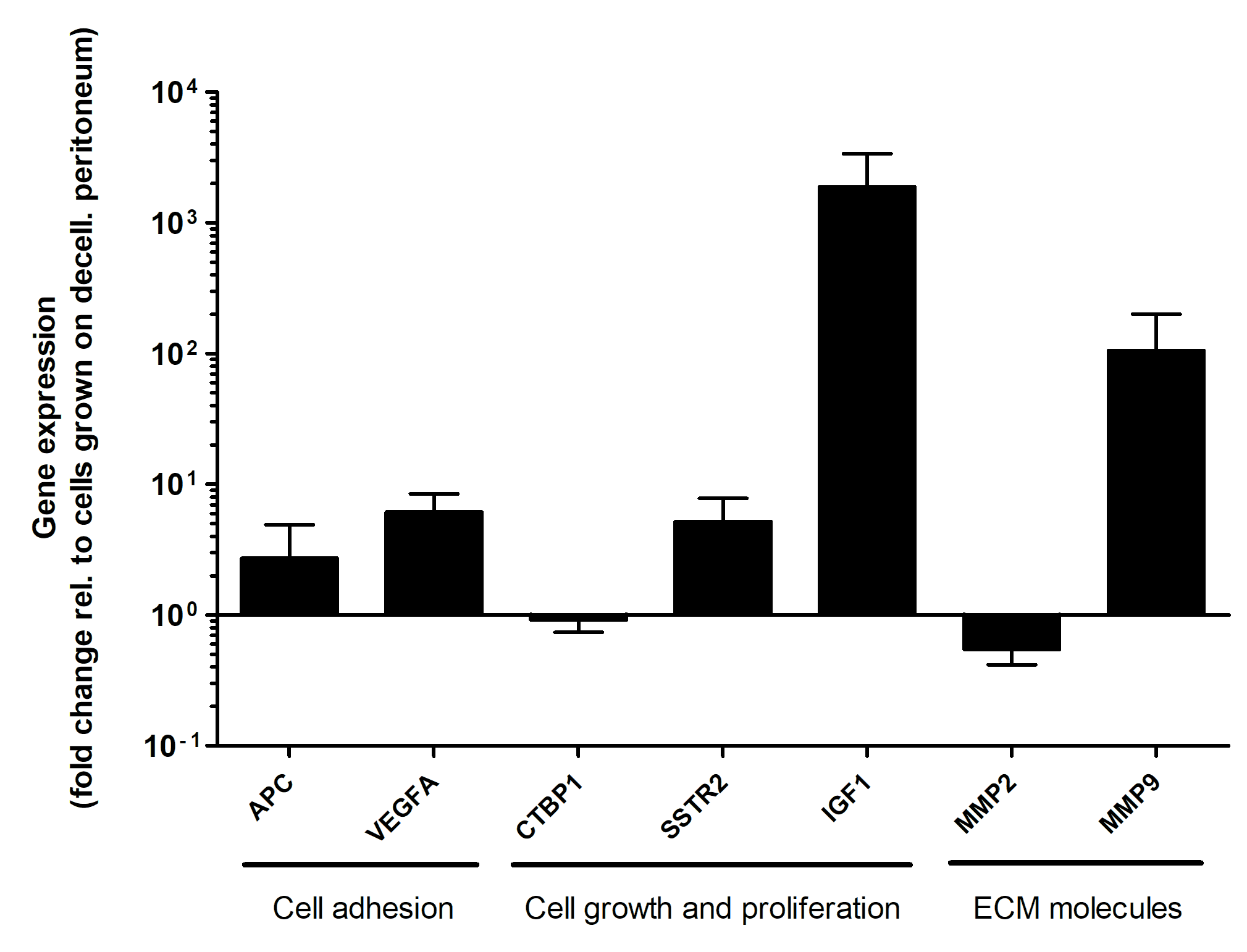

Supplement: Supplementary file 1 [file cancers-14-03760-s001.zip › S1.tif]

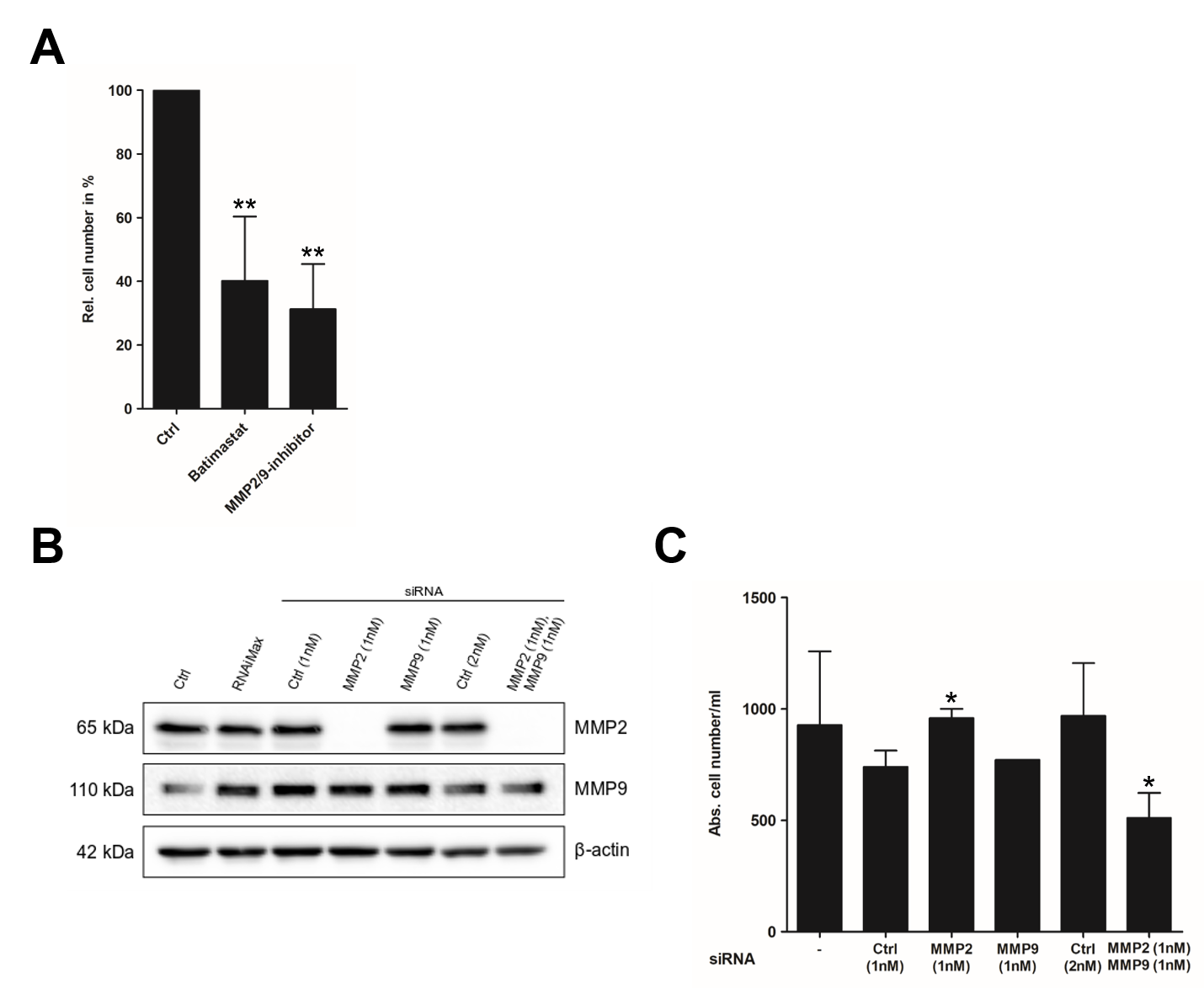

Supplement: Supplementary file 1 [file cancers-14-03760-s001.zip › S2.tif]
